# Unique Loss of the PYHIN Gene Family in Bats Amongst Mammals: Implications for Inflammasome Sensing

**DOI:** 10.1038/srep21722

**Published:** 2016-02-24

**Authors:** Matae Ahn, Jie Cui, Aaron T. Irving, Lin-Fa Wang

**Affiliations:** 1 Programme in Emerging Infectious Diseases, Duke–National University of Singapore Medical School, Singapore

## Abstract

Recent genomic analysis of two bat species (*Pteropus alecto* and *Myotis davidii*) revealed the absence of the PYHIN gene family. This family is recognized as important immune sensors of intracellular self and foreign DNA and activators of the inflammasome and/or interferon pathways. Further assessment of a wider range of bat genomes was necessary to determine if this is a universal pattern for this large mammalian group. Here we expanded genomic analysis of this gene family to include ten bat species. We confirmed the complete loss of this gene family, with only a truncated *AIM2* remaining in one species (*Pteronotus parnellii*). Divergence of the PYHIN gene loci between the bat lineages infers different loss-of-function histories during bat evolution. While all other major groups of placental mammals have at least one gene member, only bats have lost the entire family. This removal of inflammasome DNA sensors may indicate an important adaptation that is flight-induced and related, at least in part, to pathogen-host co-existence.

Unique amongst mammals, bats, of the order Chiroptera, are the only ones capable of sustained and powered flight. They account for over 20% of all classified mammalian species worldwide and are divided into two suborders Yinpterochiroptera and Yangochiroptera^1^. Bats have evolved several unique biological features including long life span^2^, low rate of tumorigenesis^3^ and asymptomatically hosting many highly pathogenic zoonotic viruses^4^,^5^, the molecular mechanisms of which are currently unknown. As inflammasome pathways have recently been recognized to be a central player in ageing, cancer and infection^6^,^7^,^8^, how these pathways in bats may differ from those of other mammals is of great importance. Our previous genomic analysis^9^ of the two bat species (*P. alecto* and *M. davidii*) revealed an absence of the PYRIN and HIN domain (PYHIN) gene family, consisting of immune sensors of intracellular DNA recently identified to activate inflammasome and/or interferon pathways^10^. As a microbial or viral signal, DNA can trigger a protective response to the invasion of pathogens, however, aberrant detection of self-DNA can trigger excessive inflammation or autoimmunity^11^. The cytokine secretion and systemic inflammation triggered by inflammasome activation must be tightly controlled, as excessive activation can lead to collateral damage and immune pathology^12^. PYHIN proteins are characterized by possessing an N-terminal PYRIN domain and mostly one or two C-terminal HIN domains^13^. All family members are located in the PYHIN gene locus which is flanked by the *SPTA1* (spectrin, α, erythrocytic 1) and *CADM3* (cell adhesion molecule 3) genes^10^.

Recently, there has been a surge in the availability of whole genome sequences for bats, with currently ten genomes released covering five different families of both Yinpterochiroptera and Yangochiroptera suborders. These ten bat species contain representatives across four of five major bat lineages: family Pteropodidae (*Pteropus vampyrus, Pteropus alecto* and *Eidolon helvum*) and superfamily Rhinolophoidea (*Rhinolophus ferrumequinum* and *Megaderma lyra*) under suborder Yinpterochiroptera; superfamily Vespertilionoidea (*Myotis lucifugus*, *Myotis davidii*, *Myotis brandtii* and *Eptesicus fuscus*) and superfamily Noctilionoidea (*Pteronotus parnellii*) under suborder Yangochiroptera. Previous transcriptome analysis of *P. alecto* revealed that bat contains and expresses all the major classes of immune genes, indicating the components of the innate and adaptive immune systems were conserved compared with other mammals^14^. Therefore, the absence of the PYHIN gene family in the two bats stands out as a major genetic change requiring vigorous assessment of a wider range of bat genomes. We hypothesize that this loss is universal among other members of this large mammalian group. As such, we further characterized the PYHIN gene locus in the ten available bat genomes and identified that while the locus was present, the specific PYHIN genes had been removed during evolution. This has ramification for sensing intracellular DNA and activation of inflammasomes.

## Results

### Loss of PYHIN genes in ten bat genomes

**Figure 1:**
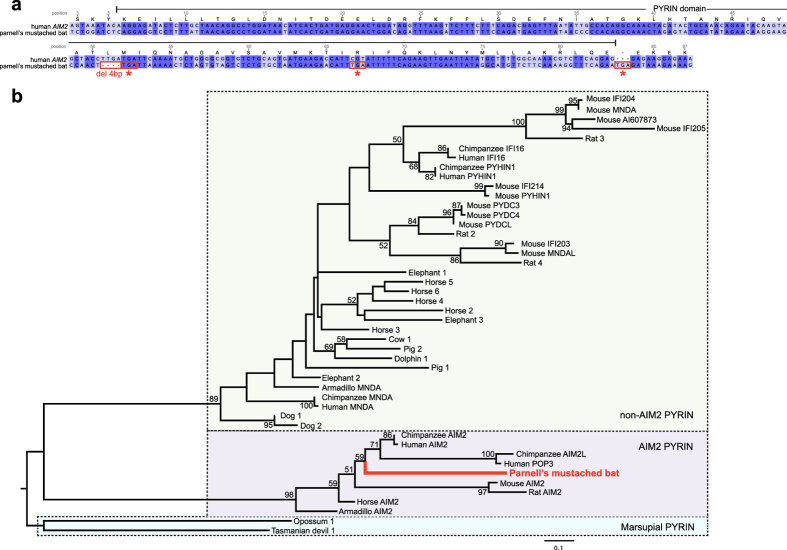
Evolution of the partial bat *AIM2* and the PYHIN PYRIN domains. (**a**) Nucleotide sequence alignment comparing the partial *AIM2* identified in the Parnell’s mustached bat genome to the PYRIN domain of human *AIM2*. A frameshift mutation due to a four-base-pair deletion and several premature stop codons (asterisks) indicate the loss of the PYRIN domain function in the bat. Identical nucleotides are highlighted in dark blue and deleterious mutations are shown in red boxes. Human AIM2 protein sequence positions were labeled above the alignment. (**b**) Phylogenetic tree of PYRIN domains from PYHIN proteins across mammalian groups. The bat PYRIN sequence is highlighted in red. The tree was rooted with marsupial sequences. Dotted boxes indicate the three distinct clades containing marsupial PYRIN, AIM2 PYRIN and non-AIM2 PYRIN respectively. Bootstrap values below 50% are not shown and branch lengths are drawn to a scale of amino acid substitutions per site.

A rigorous tblastn search using full-length PYHIN proteins from human and horse yielded no trace of PYHIN genes in nine of ten bat genomes. Unexpectedly, a partial sequence matching the first coding exon of AIM2 (absent in melanoma 2) (E-value = 6e–12; GenBank number AWGZ01046019) was revealed in the genome of Parnell’s mustached bat (*P. parnellii*). This exon encodes the PYRIN domain of AIM2. However, no trace of the other four coding exons of the AIM2 proteins including the HIN domain was found. Importantly, acquisition of a frameshift mutation due to a four-base-pair deletion and several premature stop codons indicates that such bat PYRIN domain is no longer functional (Fig. 1a). Phylogenetic analysis of the PYRIN domains of PYHIN proteins including this bat sequence revealed three distinct clades (Fig. 1b; Supplementary Data S1): marsupial PRYIN, PYRIN domains from placental AIM2 (AIM2 PYRIN), and PYRIN domains from placental non-AIM2 proteins (non-AIM2 PYRIN). This bat PYRIN sequence clearly clustered within the clade of AIM2 PYRIN and therefore indicated the existence of an ancestral *AIM2* gene in the bat common ancestor then lost during evolution. The lack of such gene fragments in the other nine bat genomes suggests that independent episodes of loss of PYHIN gene family could have occurred during bat evolution.

The lack of similarity among non-*AIM2* PYHIN genes limits the ability of tblastn searching using their full-length sequences^10^. To compensate, we conducted searches using solely the identified PYRIN and HIN domains of the PYHIN genes from horse, cow, dolphin, pig and dog, due to their close phylogenetic relationship to bats. No hits from other PYHIN genes were detected through this approach. The PYRIN domain search returned only results belonging to other PYRIN-containing proteins, such as NOD-like receptors. The lack of HIN-domain containing sequences is not surprising, as it is unique to the PYHIN gene family. All other major marsupial (metatherian) and placental (eutherian) mammalian groups except bats have at least one PYHIN gene, with many even experiencing gene duplications(Table 1). This further suggests this gene family was under negative selection during bat speciation.

### Characterization of the bat PYHIN loci

**Figure 2:**
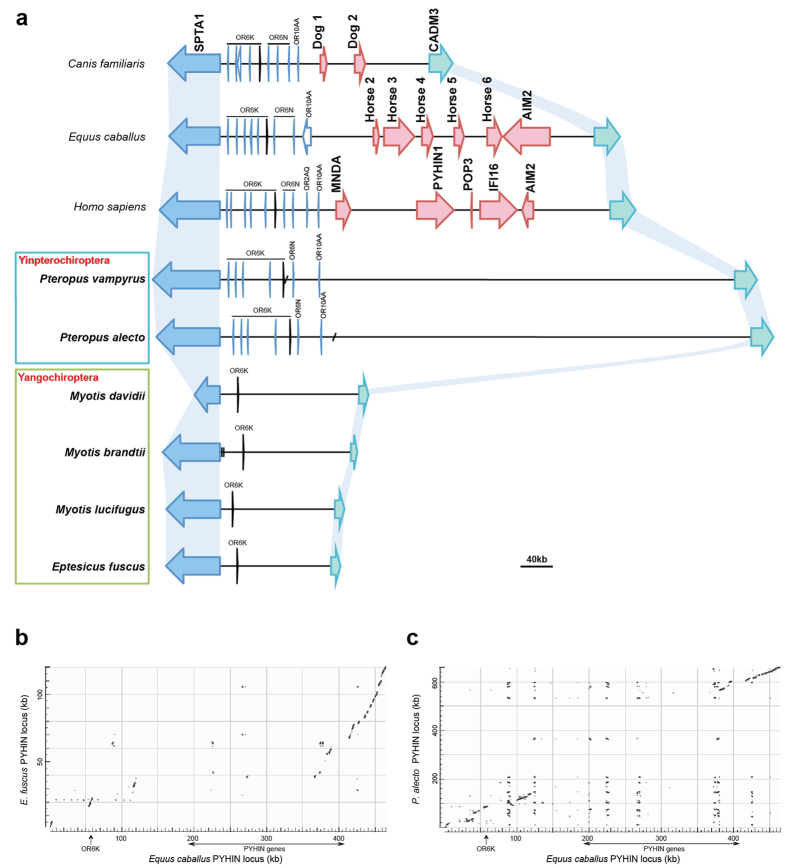
The PYHIN locus in bats and selected mammals. (**a**) Genomic characterization of the PYHIN locus in bats and selected mammalian species. The common boundaries of the PYHIN locus are defined by the *SPTA1* gene (blue) and the *CADM3* gene (green) at the two ends. Big red arrows represent the members of the PYHIN gene family. PYHIN genes from horse or dog are arbitrarily named. Short diagonal lines indicate gaps in the two bats of Yinpterochiroptera that are bridged by each other’s scaffolds. Vertical lines in *M. brandtii* indicate an inter-scaffold gap. Other bat loci lie on a single scaffold. Olfactory receptor (OR) genes or pseudogenes (light blue) are also found to cluster at one end of the locus. ORs are labeled according to their family and subfamily using the HORDE system. OR6K6 homolog in black was identified across all the loci presented here. A scale bar is presented below the figure. (**b**,**c**) Pairwise sequence comparisons of the PYHIN locus between horse and *E. fuscus* or *P. alecto* as revealed by dot-plot analysis. On the X-axis is the horse PYHIN locus and on the y-axis is the locus of *E. fuscus* (**b**) or *P. alecto* (**c**) on the kilobase (kb) scale. The OR6K6 site and the region spanning the PYHIN gene cluster are indicated.

To understand the evolution of this gene family, we performed genomic characterization of the bat PYHIN loci. We first identified the two genes flanking the bat PYHIN locus to determine its boundaries. Despite the loss of PYHIN genes, both flanking genes *SPTA1* and *CADM3* were identified in all ten bat species. We then constructed the PYHIN locus from the six bat genomes with higher coverage and/or bigger scaffold size. The PYHIN locus from genomes with lower coverage (*E. helvum, R. ferrumequinum, M. lyra* and *P. parnellii*) failed to assemble due to the small scaffold size and a lack of conserved genes or sequences identified within the region. The genomic region between the two flanking genes was retrieved from the six bat genomes and compared to the corresponding region in human, horse and dog (Fig. 2a). For *P. alecto* and *P. vampyrus*, the two flanking genes were located on two separate scaffolds. The gap between the two scaffolds was near or within the olfactory receptor cluster closer to *SPTA1* gene. Alignment of the genomic region between the two Yinpterochiroptera bats showed more than 98% identity and the gaps can be bridged by each other’s scaffold spanning the gap. For the four bats of Yangochiroptera, both flanking genes were located on a single scaffold except *Myotis brandtii*. The information of these bat genome assemblies are summarized in Table 2.

### Differences in the PYHIN locus among bats

Interesting to note, there is considerable variation in the size of the PYHIN locus (Fig. 2a). Despite the loss of the PYHIN genes within this region, both Yinpterochiroptera bats (*P. alecto* and *P. vampyrus*) have retained a PYHIN locus of greater size in comparison to those in human, horse and dog, each with five, six and two PYHIN genes identified, respectively. In contrast, all four Yangochiroptera bats, belonging to Vespertilionoidea, have a considerably shortened locus, approximately one third the size of that in human or horse.

Pairwise sequence comparisons revealed a locus homology among the four Yangochiroptera bats, with the three *Myotis* bats sharing approximately 95% identity to each other and the *E. fuscus* sharing approximately 90% identity with the *Myotis* bats. Locus comparison between the Yangochiroptera and human or horse showed large-scale contractions in the Yangochiroptera, corresponding to the regions of the PYHIN gene cluster and the olfactory receptor (OR) cluster in human or horse (Fig. 2b). Members of three OR subfamilies (6K, 6N and 10AA) were identified and clustered at one end of the locus in non-bats. However, all the ORs except for the OR6K6 homolog were found to be missing in the Yangochiroptera bats. In contrast, locus comparison between the two Yinpterochiroptera bats and non-bats revealed homology at both ends of the locus, with the exception of the PYHIN gene cluster in human or horse (about 250 kb) and the large central region (about 400 kb) in the two bats (Fig. 2c). All three OR subfamilies were identified in these two bat species. These central regions in the Yinpterochiroptera bats contain a large tandem repeat array consisting of repetitive units of about 3.5 kb, spanning approximately 300 kb in both bats. The possible role of this would require further investigation. The divergence of PYHIN loci between the two bat lineages, Pteropodidae (*P. alecto* and *P. vampyrus*) and the Vespertilionoidea (the four Yangochiroptera bats), further suggests different evolutionary processes leading to gene loss rather than a single ancestral loss event.

## Discussion

While at least one PYHIN gene was identified in other major groups of marsupial and placental mammals, bats have uniquely lost the entire gene family. We confirmed such loss is a universal pattern in all ten bat genomes. Interestingly, however, a truncated, presumably non-functional *AIM2* was identified in one species – *P. parnellii*. The divergent PYHIN genomic loci between the Yinpterochiroptera and Yangochiroptera were also observed. Taken together, these findings clearly suggest that different evolutionary events for removing PYHIN genes occurred throughout the evolutionary history of this mammalian group.

Flight is considered highly metabolically “costly” and bats in flight can rapidly increase their metabolic rate up to 34 times over their resting rate^15^. Cellular by-products of metabolism, such as reactive oxygen species (ROS), can generate harmful side effects, especially oxidative DNA damage^16^,^17^. Altered DNA damage checkpoint and repair pathways were noticed in bats, possibly to overcome this, as inferred by a concentration of positively selected genes in this pathway^9^. In addition, less ROS production or more efficient scavenging of H_2_O_2_ production has been previously observed^18^. Unique loss of the entire PYHIN gene family in bats amongst mammals may therefore also indicate an important adaptation during the evolution of flight. Previous work revealed thirty-seven gene families significantly contracted amongst the two bat genomes^9^, here we confirmed that only the PYHIN gene family is lost universally in all ten bat genomes and bat-specifically or ‘uniquely’ amongst mammals. Although bats contain other cytosolic DNA immune sensors including cGAS, DDX41, LRRFIP1, STING and DAI/ZBP1, the PYHIN family is the only identified class of DNA sensors capable of activating the inflammasome^19^,^20^. Studies have shown that the PYHIN member IFI16 together with cGAS was also required for production of STING-dependent type I interferon in response to both foreign and damaged self-DNA in infections and DNA repair-deficiency disorders^21^,^22^,^23^. In addition, STING has been implicated in sensing cytosolic DNA from other cellular stress such as autoinflammatory diseases and cancers^24^. As IFI16 has been shown to interact with STING, its absence from the bat genomes may hint at further dampening of innate immunity. We hypothesized that the evolution of flight, unique to bats among mammals, may have driven the deletion of this entire gene family. This loss would consequently allow bats to limit excessive inflammatory activation and potentially attenuate type I interferon induction, triggered by PYHIN proteins, through sensing of self-DNA from DNA damage.

In addition, bats have been recognized to host and exhibit a co-evolutionary relationship with many zoonotic RNA and DNA viruses^25^. As viral genomic DNA^26^ and host DNA damage induced by RNA viral infection^27^,^28^ can activate inflammasomes, we cannot exclude the possibility that the increased or expanded exposure to these pathogens, as compared to terrestrial mammals which cannot or do not travel long distances, might have been an additional evolutionary driver for the loss of PYHIN genes. Alternatively, the abundance of such viruses detected in bats may be linked to a consequence of PYHIN deletion. Considering the emerging importance of these immune sensors, it remains possible a new DNA sensor family or a known DNA sensor with divergent function may compensate in bats. A more specialized or specific sensor of foreign DNA versus self-DNA may also have evolved. In addition to its role in autoimmunity and autoinflammation, inflammasomes have been recognized for their roles in controlling age-related chronic inflammation and the mass-inflammatory response to invading pathogens^6^,^29^,^30^,^31^. Therefore, loss of the PYHIN gene family may play a role in the long lifespans and asymptomaticity of bats to the majority of viruses. We also confirmed that bats contain all other key components of the inflammasome pathways, such as ASC, caspase-1 and interleukin-1β. How they differ functionally from their counterparts in human and other mammals will be of great interest. NLRP3, an important inflammasome sensor responsible for recognition of a variety of stimuli including ROS and viral infection, was also found under positive selection in bats^9^. Investigation into potentially altered inflammasome sensing and activation will provide more insight into the overall process of inflammatory regulation in bats in addition to the deletion of all PYHIN genes.

## Materials and Methods

### Bat genome assemblies

Table 2 shows the summary of genome assemblies of the ten bat species released in GenBank (www.ncbi.nlm.nih.gov/genbank/). These ten bat species are from five different families of both the Yinpterochiroptera and Yangochiroptera suborders. Genomes of *P. vampyrus* and *M. lucifugus* were initially sequenced to lower coverage using Sanger sequencing (2.6× *for P. vampyrus* and 1.7× for *M. lucifugus*), but new versions with improved coverage and scaffold size are now available. The rest are sequenced using illumina Hiseq system with varied genome coverage.

### Searching for the PYHIN gene family

The species and genome sequence versions we used are: human (*Homo sapiens*, GRCh38.p2), mouse (*Mus musculus*, GRCm38.p3), rat (*Rattus norvegicus*, RGSCv3.4), horse (*Equus caballus*, EquCab2.0), dog (*Canis lupus familiaris*, CanFam3.1), cow (*Bos taurus*, Bos_taurus_UMD_3.1.1), pig (*Sus scrofa*, Sscrofa10.2), chimpanzee (*Pan troglodytes*, panTro4), elephant (*Loxodonta africana*, loxafr3.0), dolphin (*Tursiops truncates*, turTru2), armadillo (*Dasypus novemcinctus*, dasNov3), opossum (*Monodelphis domestica*, MonDom5) and Tasmanian devil (*Sarcophilus harrisii*, Devil_refv7.0). Representative genomes from major marsupial and placental mammal groups with genome assembly coverage of at least 6 times were selected for this study.

To rigorously search for PYHIN genes in the bat genomes, we first identified and obtained the PYHIN protein sequences from mammals closely related to bats within Laurasiatheria (dolphin, horse, cow, pig and dog). Recent phylogenomic analyses placed bats (order Chiroptera) as a sister group to a large clade of cetaceans, ungulates and canivores^1^. We used amino acid sequences of the five human PYHIN proteins (AIM2, IFI16, PYHIN, MNDA and POP3) containing all PYRIN and HIN domain subtypes as queries to conduct tblastn search against these genome assemblies. For those that have not been annotated accordingly in NCBI (http://www.ncbi.nlm.nih.gov/) or Ensembl (http://www.ensembl.org/), we annotated these PYHIN genes by mapping the tblastn output sequences with expressed sequence tags (ESTs) and searching the Ensembl *ab initio* predicted proteins. All PYHIN genes identified invariably reside within the PYHIN gene locus. Identified PYHIN proteins were searched for the boundaries of conserved PYRIN (PF02758) and HIN (PF02760) domains using profile hidden Markov models from the Pfam database^32^. A similar search was performed to identify PYHIN genes in all the representative genomes from major marsupial and placental mammal groups. We determined the sizes of the family by counting the number of all the PYHIN genes identified in each genome.

To search for the bat PYHIN genes, we used all amino acid sequences of PYRIN and HIN domains from dolphin, horse, cow, pig and dog as queries to conduct tblastn search against the GeneBank whole-genome shotgun (WGS) database, with gap opening and extension penalty of 11 and 1 (Expect value < 0.001). The sequence outside the conserved domains of PYHIN genes is highly variable and therefore was not used in the search. As AIM2 is the only PYHIN protein with orthologs across many species, we also used full-length horse AIM2 protein sequence together with five human PYHIN proteins as queries for tblastn search. To confirm the homology to PYHIN genes, sequences of tblastn hits plus flanking regions were extracted as queries to blastx search in NCBI non-redundant (NR) protein database.

### Phylogenetic analysis

A partial sequence in Parnell’s mustached bat was identified in tblastn searching, which was matched to the PYRIN domain of human and horse AIM2. This sequence was translated, with indels and premature stop codons removed, and aligned with other non-bat PYRIN protein sequences (Supplementary Data S1) using MUSCLE^33^. Maximum likelihood (ML) phylogenetic tree was generated using PhyML 3.0^34^. The best-fit model JTT+I+Γ was determined by ProtTest 2.4^35^. Subtree pruning and regrafting (SPR) algorism was used to search tree space, with 1,000 bootstrapping replicates applied.

### Characterization of the PYHIN loci in bat genomes

We used the flanking human *SPTA1* and *CADM3* genes as queries to identify boundaries of the PYHIN locus in the ten bat genomes. Sequences between these two gene homologs in bats were extracted for further analysis. Certain subfamilies of olfactory receptor (OR) genes and pseudogenes were found to cluster at the *SPTA1* end of the human PYHIN locus. We thus annotated these OR genes by searching these identified bat PYHIN regions against human and dog OR libraries in the HORDE database (http://genome.weizmann.ac.il/horde/). We conducted a similar search of PYHIN domains and genes within these identified PYHIN regions using tblastn. We also looked for any other non-annotated genes in these regions by performing gene structure predictions via GENSCAN (http://genes.mit.edu/GENSCAN.html) and searching the NCBI non-redundant (NR) protein database. Additionally, the PYHIN gene loci of human and two other closely related species, horse and dog, were characterized. Dot-plots comparing the loci were generated using NCBI blastn.

## Additional Information

**How to cite this article**: Ahn, M. *et al.* Unique Loss of the PYHIN Gene Family in Bats Amongst Mammals: Implications for Inflammasome Sensing. *Sci. Rep.* **6**, 21722; doi: 10.1038/srep21722 (2016).

## Supplementary Material

Supplementary Information

## Figures and Tables

**Table 1:** Summary of PYHIN gene family sizes of the major mammalian groups.

Clade	Superorder	Order	Common name	PYHIN gene number
Metatheria	Ameridelphia	Didelphimorphia	Opossum	1
	Australidelphia	Dasyuromorphia	Tasmanian devil	1
Eutheria	Xenarthra	Cingulata	Armadillo	2
	Afrotheria	Proboscidea	Elephant	3
	Laurasiatheria	Artiodactyla	Dolphin	1
			Cow	1
			Pig	2
		Perissodactyla	Horse	6
		Carnivora	Dog	2
		**Chiroptera**	**Bat**	0
	Euarchontoglires	Rodentia	Mouse	13
			Rat	4
		Primates	Chimpanzee	5
			Human	5

**Table 2:** Summary of genome assemblies of the ten bat species.

Suborder	Family	Species	Common name	Genome assembly		
				Coverage	ScaffoldN50	Version
Yinpterochiroptera	Pteropodidae	*Pteropus vampyrus*	Large flying fox	188×	5,954 kb	Pvam_2.0
		*Pteropus alecto*	Black flying fox	110×	15,955 kb	ASM32557v1
		*Eidolon helvum*	Straw-colored fruit bat	18×	28 kb	ASM46528v1
	Rhinolophidae	*Rhinolophus ferrumequinum*	Greater horseshoe bat	17×	21 kb	ASM46549v1
	Megadermatidae	*Megaderma lyra*	Greater false vampire bat	18×	17 kb	ASM46534v1
Yangochiroptera	Vespertilionidae	*Myotis davidii*	David’s myotis	110×	3,454 kb	ASM32734v1
		*Myotis brandtii*	Brandt’s bat	120×	3,226 kb	ASM41265v1
		*Myotis lucifugus*	Little brown bat	7×	4,293 kb	Myoluc2.0
		*Eptesicus fuscus*	Big brown bat	84×	13,455 kb	EptFus1.0
	Mormoopidae	*Pteronotus parnellii*	Parnell’s mustached bat	17×	23 kb	ASM46540v1
