# Supplementary material for: Unique Loss of the PYHIN Gene Family in Bats Amongst Mammals: Implications for Inflammasome Sensing
Source: Sci Rep. 2016 Feb 24;6:21722. doi: 10.1038/srep21722 (PMC4764838; doi:10.1038/srep21722)
Supplement: Supplementary Information [file srep21722-s1.pdf]

Supplementary Information

for

Unique Loss of the PYHIN Gene Family in Bats Amongst Mammals: Implications for Inflammasome Sensing

Matae Ahn, Jie Cui, Aaron T. Irving & Lin-Fa Wang*

Programme in Emerging Infectious Diseases, Duke–National University of Singapore Medical School, Singapore

* Corresponding author

Email: linfa.wang@duke-nus.edu.sg (L-FW)

Supplementary Data S1. PYRIN domain alignment sequences

>Armadillo_AIM2
KEILLLKGLDDITDEELQKFKFFLRDVLKSSTGKLEKANRTEVADLMIQNAGG
VSALDKTIAIFQKL-NYMHQAKILK-
>Armadillo_MNDA
KKIVLLKGFEAISDYHFNTIKSLLASDLKLTKKMQDDYDRIKIADLMEKKFPG
PTCVDKLIELLKDIPSLKDIVETLRK
>Chimpanzee_AIM2
KEILLLTGLDNITDEELDRFKFFLSDEFNIATGKLHTANRIQVANLMIQNAGAV
SAVMKTIRIFQKL-NYMLLAKRLQE
>Chimpanzee_AIM2l
KEILLLTSLDNITDEELDRCKCFLPDDFNIATGKLHTGNSTSSQLDLKRWRGV-
-
CSEEDGIFQKL-NYMLVAKCLRE
>Chimpanzee_IFI16
KNIVLLKGLEVINDYHFRMVKSLLSNDLKLNLKMREEYDKIQIADLMEEKFR
GDAGLGKLIQIFKDIPTLEDLAETLKK
>Chimpanzee_MNDA
KKILLLKGFELMDDYHFTTIKSLLAYDLGLTTKMQEEYNRIKITDLMEKKFQG
VACLDKLIELAKDMPSLKNLVNNLRK
>Chimpanzee_PYHIN1
KKIVLLKGLEVINDYHFRIVKSLLSNDLKLNPKMKEEYDKIQIADLMEEKFPG
DAGLGKLIEFFKEIPTLGDLAETLKR
>Cow_1
KKIVLLKGFQHINDKCFNMIKSLLAHDLKLTRKMQDEYDRVKIADLMELKFR
GPDCVDKLIELVKEIDEIKDLAKVLRK
>Dog_1
KKIILLKGLEPINDYQFSIVKSVLARDLGLTANMQEEYNKVKIADLMEKKFPG
VACVNKLIDLFKEMPSLTDIVKQLRN
>Dog_2
KKIILLKGLEPINDYQFSIVKSVLARDLGLTANMQKEYNKVKIADLMEKKFPG
VACVNKLIDLFKEMQDCEDI------
>Dolphin_1
KRIVLLKGFQHIKDYQFSMIKSLLARDLRLTSKMQDDYDRIKIADLMEVRYPG
AACVEKLIDLIKDIEELKDLAKTLRK
>Elephant_1
KKIVLLKGLEEINDYHFYIVKSLLAHDLRLXPKVQEEHDTIKIADLMAEKFPGI
ACVDKLTELLKDIKDL---------
>Elephant_2
KKIVLLKGLESINDYQFNTIKSLLARDLQLTKKMQDEYDRIKIADLMEEKFRG
AASVDKLIELFKDMPQLKDLAKSLR-
>Elephant_3
KKILLLKGLQPISEYQFNMVKSVLAHDLQLNKKAQYGYNKIQIADLMEDKFP
GATCLDILIELFEDIEGLKDLAKTLR-
>Horse_AIM2
KEILLLTGLDNITDEELDRFKFFLPDELKVSTAKLENANRTEVANLMIQNVGA
VSAVTKTIHVFQKL-NYMHVAKSLQE
>Horse_2
-

KIVLLKGLEPISAYHFSMVKSLLAHGLQLTAKPQDEYSKIEIAKLMAEKYRGA
TCVDKLIDLYKDIPELKSLVKNLR-
>Horse_3
-
KIVLLKGLEHINDYYFNVVKSLLAHDLGLTTKMQDEYNRIKIAHLIEEKFQGA
ACVNKLIELLKDIPE----------
>Horse_4
KKIILLKGLEPINEYQFSMVKFLLASDLKLTRKAQNEYNRIQIADLMTENFPRA
TCVDKLIELYKDIAELKQLAKTLKN
>Horse_5
KKILLLKGLEPISEYHFSIVKSLFAKDLKLTEKAQNEYNRIQIADLMEESFPEG
ACVDALIDLYKDIAELKDLAKTLK-
>Horse_6
KRIVLLKGLEPISEYHFSIVKSLLAHDLKLSRKAQNEYNRIQIADLMEVKFRGA
ACVDALIKLYKDIAELKELAKTLKN
>Human_POP3
KEILLLTSLDNITDEELDRFKCFLPDEFNIATGKLHTLNSTS-SQLDLKRWHGV-
-CSEEDRIFQKL-NYMLVAKCLRE
>Human_AIM2
KEILLLTGLDNITDEELDRFKFFLSDEFNIATGKLHTANRIQVATLMIQNAGAV
SAVMKTIRIFQKL-NYMLLAKRLQE
>Human_IFI16
KNIVLLKGLEVINDYHFRMVKSLLSNDLKLNLKMREEYDKIQIADLMEEKFR
GDAGLGKLIKIFEDIPTLEDLAETLKK
>Human_MNDA
KKILLLKGFELMDDYHFTSIKSLLAYDLGLTTKMQEEYNRIKITDLMEKKFQG
VACLDKLIELAKDMPSLKNLVNNLRK
>Human_PYHIN1
KKIVLLKGLEVINDYHFRIVKSLLSNDLKLNPKMKEEYDKIQIADLMEEKFPG
DAGLGKLIEFFKEIPTLGDLAETLKR
>Mouse_AI607873
KRIVLLKGLECINKHHFSLFKSLLARDLSLERDNQEKYSTIQIANMMEEKFKP
DAGLGELIEFCEKVPALRKRAEILKK
>Mouse_AIM2
--
MLLLTGLDHITEEELKRFKYFALTEFQIARSTLDVADRTELADHLIQSAGAAS
AVTKAINIFQKL-NYMHIANALE-
>Mouse_IFI214
KRIVLLTGLMGINDHDFRMVKSLLSKELKLNKMQDEYDRVKIADLMEDKFP
KDAGVVQLIKLYKQIPGLGDIANKLKN
>Mouse_IFI203
KNIVLLKGLENMEDYQFRTVKSLLRKELKLTKKMQEDYDRIQLADWIEDKFP
KDAGLDKLIKVCEHIKDLKDLAKKLK-
>Mouse_IFI204
KRIVLLRGLECINKHYFSLFKSLLARDLNLERDNQEQYTTIQIANMMEEKFPA
DSGLGKLIAFCEEVPALRKRAEILKK
>Mouse_IFI205
-
RLVLLEGLECINKHQFNLFKSLMVKDLNLEEDNQEKYTTFQIANMMVKKFPA
DAGLDRLINFCERVPTLKKRAEILKK

>Mouse_MNDA
KRIVLLRGLECINKHYFSLFKSLLARDLNLERDNQEQYTTIQIANMMEEKFPA
DSGLGKLIEFCEEVPALRKRAEILKK
>Mouse_MNDAL
KKIVLLKGLESMEDYQFRTVKSLLRKELKLTKKLQEDYDRIQLADWMEDKFP
KYAGLDKLIKVCEHIKDLKDLAKKLK-
>Mouse_PYDC3
KQIVLLSGLEYMNDYNFRALKSLLNHDLKLTKNMQDDYDRIKIADLMEEKFP
EDAGLSKLIEVCEDIPELAARVDILR-
>Mouse_PYDC4
KQIVLLSGLEYMNDYNFRALKSLLNHDLKLTKNMQDDYDRINIADLMEEKFP
EDAGLSKLIEVCEDIPELAARVDILR-
>Mouse_PYDCl
KQIVLLSGLEYMNDYNFRALKSLLNHDLKLTKNMQDDYDRIKIADLMEEKFP
EDAGLSKLIEVCEDIPELADHVDILRK
>Mouse_PYHIN1
KRIVLLTGLMGINDHDFRMVKSLLSKELKLNRMQDQYDRVKIADLMEDKFP
KDAGVDQLIKLYKQIPGLGDIANKLKN
>Opossum_1
---ILLSGLEELQNEDFKKFKFFLSNDCLMMR--
VHNLDRIDLATCMKKQLPCPSFMNKFIEILKKM-NLNETAKKYK-
>Pig_1
KKIILLKGLEKIDEYHFSMIKSLLAHDLELTREMQDNYDRVKIADLMELKFPGI
TCVNILEELLSDMIK----------
>Pig_2
KRIVLLKGFQHMDDYHFKMIKSLLAHDLRLTRKMQEEYDRIKIADLMEERFR
GAACVDKLIELVKGIDDIKHLVKPLQ-
>Rat_AIM2
--
MLLLTGLDHITEEELKRFKYLALTEFNIPRKTLNIADRTELADQLIQSAGAASA
VAKAISIFQKL-NYMDIAKALE-
>Rat_2
KKIVLMFGLERINDYNFRTLKSLLTHDLKLTQKMQDDYDRIKIADLMEEKFPE
DAGLSKLIEVWEGIPEFEDHADTLRK
>Rat_3
KRIVLLKGLEPLSSHHFSLFKSLLASDLKLERHMQEQYTKVQIADMMEDEFPD
DAGLGKFIKFCEDLPALRKRAEILKR
>Rat_4
KEIVLIKGLEDMKDYAFRTIKSLLRKELNLTKKMQDDYDRIQLADLLEDKFPQ
DAGLSKLIEVCESIEELKELTDNLKR
>Tasmanian_devil_1

ILLESLTELDEEEFKICKYHLKNRGLIIRMEGKGDRTDIAESMKAKFGAVSALK
HITNVFKKMNLNEIVENLQ-
>Parnell’s_mustached_bat
QEVLLLTGLDNITDEELDRFKIFFPDEFITPTGKLEYAYRTR--
KPTIKNSSVVSVLMKNI-IFQKL-NYRHVLQKVSE
